# A Decade of Partnerships and Progress in Pathogen Genomics in Public Health Practice

**DOI:** 10.3201/eid3113.241670

**Published:** 2025-05

**Authors:** Duncan MacCannell, Bronwyn MacInnis, Scott Santibanez, Margaret A. Honein, Wendi Kuhnert, Christopher Braden

**Affiliations:** Centers for Disease Control and Prevention, Atlanta, Georgia, USA (D. MacCannell, S. Santibanez, M.A. Honein, W. Kuhnert, C. Braden); The Broad Institute of MIT and Harvard, Cambridge, Massachusetts, USA (B. MacInnis).

**Keywords:** Public health, genomics, pathogens, SARS-CoV-2, viruses

The field of public health is evolving continuously, driven in part by advances in science and biotechnology. Among the most transformative of those changes are the rapid acceleration of genomics, bioinformatics, and molecular epidemiology throughout the public health system and the growing list of applications of such methods for infectious diseases of both public health and economic concern (*1*). Those technologies have revolutionized the understanding, tracking, and prevention of many infectious diseases and are notable for their adaptability to different pathogens and changing diagnostic, surveillance and response requirements, as well as to different levels of technical capacity in laboratory environments where they are in use.

To understand the effect of pathogen genomics on public health, we must consider the types of materials that can be sequenced, which include pathogens (viral, bacterial, fungal, parasitic) from host, vector, and environmental samples; the unique and complex features of pathogen genomes; and the thousands of potential datapoints their genomes can encode. Because molecular techniques generate massive amounts of raw sequence data, investments in bioinformatics, including data analysis tools, computing infrastructure, and skilled workforce, are critical to translate pathogen sequence data into actionable public health information. By combining new types of data with traditional epidemiologic approaches, sequencing provides an incredibly powerful new set of tools for investigating the spread and informing the prevention and control of infectious diseases.

The SARS-CoV-2 pandemic led to pathogen genomic sequencing being used at a previously unfathomable scale. By the end of 2024, >17 million virus genomes were catalogued globally, roughly one third from laboratories in the United States. Those sequences were assembled, annotated, and shared through several major international sequence repositories, including GenBank (*2*) and the GISAID (https://www.gisaid.org) EpiCoV database (*3*). This rapid sharing of pathogen genomic data enabled the global public health community to monitor and respond to the evolution and transmission of the virus and to begin critical work on technical and ethical requirements for better and more equitable data sharing.

Within the United States, SARS-CoV-2 sequence data provided a critical national baseline for variant surveillance; enabled reference characterization and risk assessment of emerging variants; aided in ongoing assessment of approved diagnostics, vaccines and therapeutics; and guided overall pandemic response strategy and resource prioritization (*4*). The data also provided necessary insights at the state and local level, enabling many jurisdictions to implement comprehensive surveillance for unusual variants, phenotypes, or clinical outcomes; to guide clinical and therapeutic strategy; and to enhance outbreak response and infection control efforts on the basis of genomic and phylodynamic information (*5*).

For more than a decade, the Centers for Disease Control and Prevention (CDC) Advanced Molecular Detection (AMD) program has been at the forefront of integrating genomics and other novel laboratory technologies into routine public health practice. AMD is a cross-cutting program that works across CDC’s infectious disease centers, state and local public health departments, and a growing network of academic, commercial, and global partners to help drive innovative, high complexity laboratory technologies and the bioinformatic and workforce changes needed to implement the technologies effectively, sustainably, and at scale.

Initial AMD investments focused on building sequencing and bioinformatics capacity for CDC core programs, while expanding capacity in state and local public health departments. More recent activities have sought to strengthen and build the public health workforce and create opportunities for academic and private sector collaboration. Future efforts will focus on promoting public engagement around AMD technologies, developing a professionally diverse and resilient technical workforce, ensuring representativeness of AMD studies and leadership, and expanding access to the benefits of these investments. Global investments and partnerships in pathogen genomics have already shown great promise in building contextually relevant genomic sequencing and analysis capacity at a local and regional level in many low-to-middle income countries, including establishing locally managed networks of infrastructure, resources and expertise.

This supplemental issue of *Emerging Infectious Diseases* brings together a collection of articles that showcase the progress and effect of pathogen genomics, bioinformatics, and genomic epidemiology on public health, illustrating both the current effects and the future promise of such technologies. The technologic contributions in this supplement issue highlight a range of different applications and collaborations, including case studies of outbreak responses and surveillance programs, necessary advances in bioinformatics tools and databases, practical considerations for capacity building and benefit to populations experiencing greater illness and death, and perspectives on the future direction of the field.

As public health continues to evolve and to adapt to new threats and challenges, the integration of new technologies and the data they provide will continue to shape the landscape of infectious disease surveillance and response. The insights gained from those new data will be crucial in addressing emerging and reemerging infectious diseases, ensuring we are better prepared to face public health challenges today and tomorrow.
